# Febrile Neutropenia as the First Manifestation of T-Cell Large Granular Lymphocytic Leukemia

**DOI:** 10.7759/cureus.31274

**Published:** 2022-11-08

**Authors:** Cristiana Honrado Martins, Luís Neves da Silva, Vânia Gomes, Guilherme Castro Gomes

**Affiliations:** 1 Oncology, Hospital de Braga, Braga, PRT; 2 Internal Medicine, Hospital de Braga, Braga, PRT

**Keywords:** lymphoproliferative disorders, hematology, febrile neutropenia, elderly, t-cell large granular lymphocytic leukemia

## Abstract

T-cell large granular lymphocytic (T-LGL) leukemia is a rare lymphoproliferative disorder, characterized by peripheral blood and bone marrow infiltration with large granular lymphocytes (LGL), splenomegaly, cytopenias, and a frequent association with autoimmune diseases. Recurrent bacterial infections due to neutropenia are the main reason why patients come to medical attention. Despite not being a curable disease, T-LGL leukemia usually has an indolent course, with deaths mainly resulting from severe infections. Treatment is often not required, however, when needed, aims to relieve symptoms, and reduce infections and transfusion needs.

We describe a case of an 86-year-old female patient with febrile neutropenia, diagnosed with T-LGL leukemia after the resolution of infection and exclusion of other causes of neutropenia. A “*watch and wait”* approach was established after a multidisciplinary discussion.

This case shows a frequent presentation of a rare disease, as well as the approach from diagnosis to treatment, reminding clinicians that T-LGL leukemia should be considered in the differential diagnosis of adults with febrile neutropenia.

## Introduction

T-cell large granular lymphocytic (T-LGL) leukemia is a rare lymphoproliferative disorder, usually with an indolent course, characterized not only by peripheral blood and bone marrow infiltration with large granular lymphocytes (LGL), splenomegaly, and cytopenias but also by a frequent association with other hematologic and autoimmune diseases, particularly rheumatoid arthritis [1]. T-LGL leukemia affects both sexes equally, with a median age of 60 years [1]. Recurrent bacterial infections, as a consequence of neutropenia, is the main reason why patients require medical attention [2].

In this report, we will present a case of an elderly female patient with febrile neutropenia who was diagnosed with T-LGL leukemia after the resolution of infection and exclusion of other causes of neutropenia. 

## Case presentation

An 86-year-old woman with a past medical history of type 2 diabetes mellitus, dyslipidemia, Parkinson’s disease, peripheral artery disease, and severe osteoarticular degenerative disease, which caused a permanent need for help in activities of daily living, was admitted to the emergency department with week-long complaints of prostration and liquid dejections, without vomiting or nausea. She also had a fever (peak of 38.3ºC), which responded to antipyretics, and denied sick contacts. On physical examination, signs of dehydration were evident, pulmonary and cardiac auscultations were normal, the abdominal palpation did not present tenderness, masses, or organomegaly and there was no cutaneous rash or other visible lesions, as well as no palpable peripheral adenopathies. 

The blood panel showed leukopenia with neutropenia (absolute neutrophils count of 0.3x10^3^/μL) and lymphocytosis that was not previously reported (absolute lymphocyte count of 5.2x10^3^/μL). There was no anemia or an abnormal platelet count. C-reactive protein was elevated (88 mg/L). The chest X-ray showed a right pulmonary base hypotransparency.

She was admitted to the internal medicine ward, with the diagnosis of febrile neutropenia and right-sided pneumonia. Empirical treatment with piperacillin/tazobactam 4g/0.5g, every six hours was initiated. Blood and sputum cultures collected before the start of antibiotic therapy were sterile, and the urinary antigens screening for *Legionella pneumophila* and *Streptococcus pneumoniae *were also negative. Because of the complaints of liquid dejections, stool tests were performed, which returned negative for *Clostridium difficile*, adenovirus, and rotavirus. Microbiological and parasitological fecal cultures were also negative. The diagnosis of viral gastroenteritis was assumed, and the patient’s bowel movements normalized on the first day of hospitalization.

Concerning the patient's previously unknown granulocytic lineage alterations, the differential diagnosis of infectious, neoplastic, and iatrogenic causes was considered.

Despite the most plausible hypothesis of bone marrow suppression as a reaction to infection, leukopenia with neutropenia (absolute neutrophils count of 0.3x10^3^/μL in repeated hemograms) persisted after seven days of antibiotic treatment, clinical improvement, and inflammatory parameters resolution. Her usual neurological and psychotropic medication (such as clonazepam and carbidopa/levodopa) was discontinued, despite the rarity of described cases with such side effects. No neoplasia was clinically apparent.

**Figure 1:**
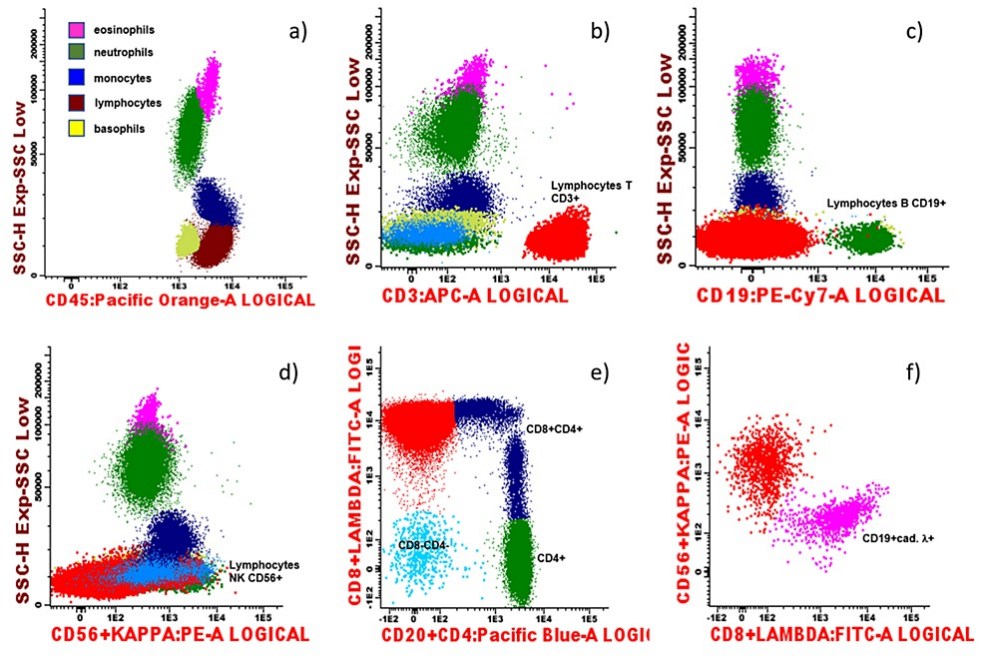
Flow cytometry charts - primary characterization (a) CD45 is a receptor-type protein tyrosine phosphatase ubiquitously expressed in all nucleated hematopoietic cells. In this patient's sample, we have all the normal leukocytes differentiation subtypes represented. Please note the marked neutropenia; (b) CD3+ T lymphocytes (red colored population) are increased, being a marker of B type leukemia/lymphoma differentiation; (c) this chart shows a normal range of B lymphocyte cells population; (d) this chart shows a normal range of natural killer (NK) CD56+ lymphocyte population, with normal receptors expression; (e) this chart shows a pathologic CD8+/CD4+ ratio, with an increased CD8+ T cell population, which led to further immunophenotyping investigation (as detailed in figure 2); (f) this chart shows a normal kappa/lambda expression of B lymphocyte cells.

**Figure 2:**
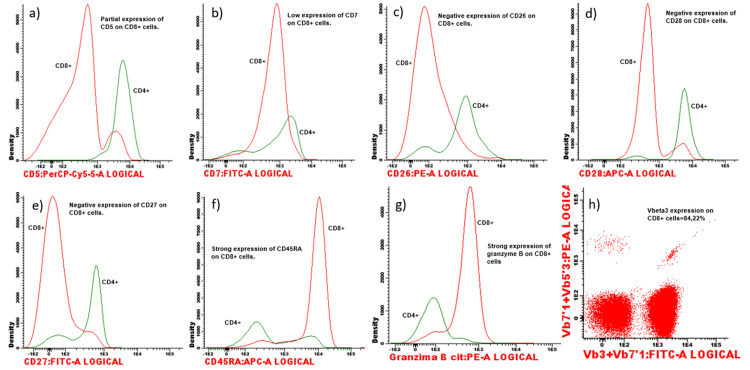
Flow cytometry charts - Further investigation (a) This chart shows a partial CD6 expression in CD8+ cells; (b) this chart shows a weak CD7 expression in CD8+ cells; (c) this chart shows a negative CD26 expression in CD8+ cells; (d) this chart shows a negative CD28 expression in CD8+ cells; (e) this chart shows a negative CD27 expression in CD8+ cells; (f) this chart shows a strong positive CD24RA expression in CD8+ cells; (g) this chart shows a positive cytotoxic Granzyme A expression in CD8+ cells; (h) this chart shows a Vbeta3 expression of 84.22% in CD8+ T cells, showing a monoclonality for T-cell receptor (TCR) Vbeta3 area, typical in chronic leukemias. The presented immunophenotype is typical of CD8+ T-cell large granular lymphocytic (T-LGL) leukemia.

Further investigation revealed a serum protein electrophoresis with a gamma polyclonal band (elevated immunoglobulin A and G) and no monoclonal peak; a serum-free light chains elevation, both kappa, and lambda, with a normal ratio; immunological scars of old and multiple viral infections (cytomegalovirus (CMV), Epstein-Barr virus (EBV), herpes virus, and adenovirus). A peripheral blood smear showed a few pleomorphic lymphocytes and platelet anisocytosis. Blood immunophenotyping revealed marked reactive neutropenia, normal populations of B and natural killer (NK) lymphocytes, and monoclonal CD8+ T cells, as can be seen in the flow cytometry charts of Figure 1A-1F and Figure 2A-2H, making T-LGL leukemia the most probable diagnosis.

This case was discussed with the oncology-hematology team, and a *“watch and wait”* approach was decided as the best treatment plan for this patient.

## Discussion

This clinical case illustrates one of the most frequent presentations of T-LGL leukemia. Febrile neutropenia is a well-known condition in patients treated with chemotherapy and immunosuppressive treatments. However, in patients who are not in those conditions, the etiology of neutropenia can be difficult to diagnose as the differential diagnosis is broad. Therefore, in our patient, when the hypothesis of bone marrow suppression due to the acute infection was starting to look less likely, an exhaustive investigation was performed which ultimately led to the diagnosis of T-LGL leukemia. 

A diagnosis of T-LGL leukemia is usually based on blood studies (differential blood count, blood smear, immunophenotyping, T-cell receptor (TCR) rearrangement analysis). Bone marrow exams are not routinely recommended unless they are relevant for differential diagnosis [3]. Other causes that can lead to reactive LGL proliferation such as viral infections (e.g. human immunodeficiency virus (HIV), hepatitis B virus (HBV), hepatitis C virus (HCV), CMV, EBV), lymphomas, and solid tumors should be excluded, as was done in this patient [4].

Despite the high prevalence of autoimmune disorders in T-LGL leukemia patients, our patient did not present any symptoms or serological anomalies indicating the presence of autoimmune disease [5,6].

T-LGL leukemia is not a curable disease but usually has an indolent course, with overall survival of 70% at 10 years [4]. Deaths mainly result from severe infections [7]. Treatment, which is based on immunosuppressive drugs, is often not required as only half of patients need treatment at the time of diagnosis [4]. When needed, it aims to relieve symptoms and reduce infections as well as blood transfusion needs. Active treatment is only indicated in case of symptomatic disease or severely impaired blood cell counts: neutrophils <0.5x10^3^ cells/μL, neutropenia-associated infections, hemoglobin <10 g/dL or transfusion needs, platelets <50x10^9^ cells/L, symptomatic autoimmune disease, symptomatic splenomegaly and severe B-symptoms [4,8].

The patient in this case report would be considered a candidate for active treatment due to her persistent low neutrophil count. However, due to her frailty, a "*watch and wait"* strategy was assumed.

It should be noted that in medical literature some case reports of patients with neutropenia and recurrent infections ultimately diagnosed with T-LGL leukemia can be found [9,10]. In contrast with the patient in our case, those patients had a larger number of infections and were younger. In fact, the median age at diagnosis for T-LGL leukemia is 60 years, making this case different due to the more advanced age at diagnosis. Also, the alertness of the team who sought to investigate the unexplained neutropenia was perhaps one of the factors that led to an earlier diagnosis of T-LGL leukemia and so, avoiding the classical course of several infections before reaching the underlying diagnosis. 

## Conclusions

T-LGL leukemia is a rare disease that mainly comes to medical attention because of infectious diseases that are more frequent in these patients due to neutropenia.

This clinical case aims not only to elucidate how to manage this condition from diagnosis to treatment but also to remind clinicians to consider T-LGL leukemia in the differential diagnosis of febrile neutropenia in adult patients, especially in the elderly and if there is a coexisting history of autoimmune disease.
